# Welfare issues and potential solutions for laying hens in free range and organic production systems: A review based on literature and interviews

**DOI:** 10.3389/fvets.2022.952922

**Published:** 2022-08-05

**Authors:** Claire Bonnefous, Anne Collin, Laurence A. Guilloteau, Vanessa Guesdon, Christine Filliat, Sophie Réhault-Godbert, T. Bas Rodenburg, Frank A. M. Tuyttens, Laura Warin, Sanna Steenfeldt, Lisa Baldinger, Martina Re, Raffaella Ponzio, Anna Zuliani, Pietro Venezia, Minna Väre, Patricia Parrott, Keith Walley, Jarkko K. Niemi, Christine Leterrier

**Affiliations:** ^1^INRAE, Université de Tours, BOA, Nouzilly, France; ^2^JUNIA, Comportement Animal et Systèmes d'Elevage, Lille, France; ^3^VETOPOLE 26, Châteauneuf-sur-Isère, France; ^4^Faculty of Veterinary Medicine, Utrecht University, Utrecht, Netherlands; ^5^ILVO, Instituut voor Landbouw-, Visserij- en Voedingsonderzoek, Melle, Belgium; ^6^Department of Veterinary and Biosciences, Faculty of Veterinary Medicine, Ghent University, Ghent, Belgium; ^7^ITAVI, Nouzilly, France; ^8^Department of Animal Science, Aarhus University, Aarhus, Denmark; ^9^Thuenen Institute of Organic Farming, Westerau, Germany; ^10^AIAB, Associazone Italiana per l'Agricultura Biologica, Rome, Italy; ^11^Slow Food, Bra, Italy; ^12^Veterinari Senza Frontiere Italia, Sede c/o Istituto Zooprofilattico Sperimentale delle Venezie viale dell'Università, Padova, Italy; ^13^Natural Resources Institute Finland (Luke), Bioeconomy and Environment, Helsinki, Finland; ^14^Harper Adams University, Newport, United Kingdom; ^15^Natural Resources Institute Finland (Luke), Bioeconomy and Environment, Seinäjoki, Finland; ^16^CNRS, IFCE, INRAE, Université de Tours, PRC, Nouzilly, France

**Keywords:** poultry, organic, free range, health, hen, pullet, welfare

## Abstract

In free-range and organic production systems, hens can make choices according to their needs and desires, which is in accordance with welfare definitions. Nonetheless, health and behavioral problems are also encountered in these systems. The aim of this article was to identify welfare challenges observed in these production systems in the EU and the most promising solutions to overcome these challenges. It is based on a review of published literature and research projects complemented by interviews with experts. We selected EU specific information for welfare problems, however, the selected literature regarding solutions is global. Free range use may increase the risk of infection by some bacteria, viruses and parasites. Preventive methods include avoiding contamination thanks to biosecurity measures and strengthening animals' natural defenses against these diseases which can be based on nutritional means with new diet components such as insect-derived products, probiotics and prebiotics. Phytotherapy and aromatherapy can be used as preventive and curative medicine and vaccines as alternatives to antibiotics and pesticides. Bone quality in pullets and hens prevents keel deviations and is favored by exercise in the outdoor range. Free range use also lead to higher exposure to variable weather conditions and predators, therefore shadow, fences and guard animals can be used to prevent heat stress and predation respectively. Granting a free range provides opportunities for the expression of many behaviors and yet many hens usually stay close to the house. Providing the birds with trees, shelters or attractive plants can increase range use. Small flock sizes, early experiences of enrichment and personality traits have also been found to enhance range use. Severe feather pecking can occur in free range production systems, although flocks using the outdoor area have better plumage than indoors. While many prevention strategies are facilitated in free range systems, the influence of genetics, prenatal and nutritional factors in free range hens still need to be investigated. This review provides information about practices that have been tested or still need to be explored and this information can be used by stakeholders and researchers to help them evaluate the applicability of these solutions for welfare improvement.

## Introduction

### Alternative laying hen farming systems in Europe

Animal welfare is a major concern and its conceptualization has evolved from a simple perception of health status to also embrace a full understanding of an animal's mental state related to its environment (1). The conventional cage rearing system for laying hens was developed after the Second World War as an option to increase production. In 2012, the conventional cage was prohibited in the EU and only furnished cages and non-cage systems (including free range and organic systems) were allowed.

In 2020, the laying hens kept in the European Union (EU) produced around 116 billion eggs which was equivalent to 7.2 million tons of eggs and which represented 9.4% of the world's production (2). According to the marketing standards for eggs, (EC/589/2008), eggs can be sold as “Free range eggs,” “Barn eggs,” or “Eggs from caged hens.” The fourth category is “Organic eggs”. Eggs can be called organic only when they have been produced and controlled according to the EU organic regulation (EU/2018/848 and EU/2020/464). Depending on the farm, the production of organic eggs can also comply with the requirements for free range egg production or produced by hens housed in mobile shelters. Alternative forms of egg production include all other forms except eggs from caged hens (Table 1; Supplementary Data S1). Eggs are among the most purchased organic food products in the EU (3). The outputs of free range and organic systems are currently increasing (4) and more than 26.3 million laying hens were certified organic in the EU in 2019 (+9% compared to 2018), however these percentages vary a lot among countries: the percentage of free range hens varied from 3% in Portugal to 58% in UK in 2019 while the percentage of organic hens varied from 1% in Poland, Portugal and Spain to 16% in France (2).

**Table 1:** Characteristics of alternative rearing systems for laying hens^*^.

**Organic**		**Non-organic**	
Standard egg production	Enhanced	Low-input systems	
(EU) 2018/848	Beyond (EU) 2018/848		
(EU) 2020/464			
Free range and mobile egg production		Free range	Mobile egg production
Maximum for flock size (<3,000 hens per compartment), stocking density on the range (one hen/4 m^2^), stocking density indoors (six hens/m^2^)	Enhanced especially for: flock size, space allowance, duration of outdoor access, prohibition of beak trimming, provision of nests, perches, vegetation and shelters on the range and environmental enrichments	The birds may live indoors but must have access to outdoors (three subcategories of free-range according to the directive 543/2008/EC)	The birds live in movable shelters with access to pasture.

^*^Detailed requirements in the Supplementary Data S1.

In outdoor systems such as free range, hens can make choices according to their needs and desires, which is in accordance with most animal welfare definitions and corresponds to consumer preferences regarding farming systems (5). Moreover, animal welfare remains the main reason to buy organic (3). Nonetheless, behavioral and health problems are also encountered in these systems (6–8). Some are similar to issues associated with conventional systems, such as the culling of day-old male chicks, feather pecking and keel bone fractures, while others are primarily associated with outdoor production, such as increased risks of endo-parasites, predation and infection with avian influenza.

### Aim and approach

The aim of this article was to summarize animal welfare challenges observed in free range and organic laying hen production systems in the EU and to consider the most promising solutions to overcome these challenges. For this purpose, data related to welfare issues in laying hens were collected from multiple sources (Supplementary Data S2). Published literature and research projects' results were reviewed and key expert informants in Italy, France, United Kingdom and Finland were interviewed. The interviews took place within the PPILOW project dealing with the welfare in pigs and poultry.

In each country, the key informants included a farmer (organic or outdoors), a vet involved in organic or free range production, a representative from a breeding company and a representative from a firm involved in egg quality, a premix producer specialized in premix manufacturing and advising in animal nutrition, and a non-governmental organization involved in animal welfare (Supplementary Data S2). The objective of the interviews was to understand the major issues in welfare according to practitioners, to make sure that the literature review did not miss any issue or solution and to give a hierarchy to the issues. The information form, the consent form and the guidelines were approved by the French ethics committee Polethis from Paris-Saclay university. Information provided in the interviews was checked and balanced with published literature. Welfare issues and phenotypic traits were defined according to the Animal Health Ontology for Livestock, AHOL (Supplementary Data S3).

## Health of laying hens in free range and organic systems

Free range systems allow outdoor access and contact with infected feral, wild animals or their excreta represents a higher risk of some infections such as endo-parasitism and *Salmonella* infection. The key informants pointed out biosecurity issues in production with outdoor access. Biosecurity is defined as cumulative steps taken to keep disease from a farm and to prevent the transmission of disease within an infected farm to neighboring farms. In outdoor systems, both aspects of biosecurity are different from indoor systems since it is difficult to avoid contacts with infectious agents and to complete disinfection for example, like it is carried out indoors. Therefore, it is often mentioned that free range access results in more difficulties in keeping housing free from bacteria or viruses such as influenza found in wild birds and in higher risks of parasitism.

### Infectious diseases

#### Bacterial and viral diseases

Several key informants mentioned biosecurity and regulation issues because free range systems, as in conventional systems, are affected by food borne diseases and their regulation has wide impacts on the practices. The main pathogenic bacteria are *Mycoplasma, Pasteurella, Escherichia coli* and *Salmonella*. The occurrence of *Salmonella* spp. is highly monitored and regulated since, like *Campylobacter*, it can lead to food borne disease in humans and both bacteria can be encountered in the environment. *Salmonella* spp. is among the most common zoonotic pathogens responsible for bacterial infection that compromises food safety but not animal welfare. Its propagation in animals is limited by vaccinating layers in some EU countries since vaccination is highly effective in prevention. Organic and free range flocks are also particularly susceptible to avian influenza, a recurrent viral infection that is caused by avian influenza type A viruses, because the risk of contamination is higher in outdoor systems (9). *Salmonella*, mostly *Salmonella* Enteritidis and *Salmonella* Typhimurium (10), *Campylobacter* and avian influenza can contaminate reared flocks after direct or indirect contact with infected wild animals. Infection by *Campylobacter hepaticus* is emerging in Europe hence Spotty liver disease increase, an acute necrotic hepatitis causing mortality and falls in egg production especially in free range production (11), however it was not mentioned by key informants.

#### Parasitism

Parasitism in organic and free range egg production mainly consists of endo-parasitism, especially helminths, i.e., nematodes (*Ascaridia galli* and *Heterakis* spp. especially) and cestodes (*Raillietina, Choanotaenia, Davainea* especially) and in protozoa (*Eimeria*, causing coccidiosis). In 2011, it was concluded from an epidemiological study in Germany that the vast majority of organic laying hens were subclinically infested with at least one helminth species (12). Similarly, a study conducted in eight different European countries found that 69.5% of organic layers were infested with *Ascaridia galli* (13). The overall mean prevalence for *Heterakis* spp. was 29.0% with a large variation between countries (13). In this survey, *Raillietina* was the most widespread cestode, but it occurred at a moderate level (13.6%). This study also demonstrated that pasture-access time was negatively linked to *Ascaridia galli* worm burden, which does not support the idea that outdoor access would increase the risks of helminth infection. This also highlights the need to further investigate the complex transmission dynamics since a better understanding of the transmission routes in free ranges and their variations with wild fauna behavior would help to reduce infection in free range hens (14).

Ecto-parasitism in poultry is mainly due to red poultry mites (*Dermanyssus gallinae*). Biosecurity rules to prevent contamination are again more difficult to use in free range systems than in cages since red mites are present in wild birds and eggs and larvae are hard to destroy if wood shelters are used. Red mites are parasites that attack hens at night to get blood meals and they can induce anemia, decreased egg production and increased stress, feather pecking and mortality (15). Moreover, red mites are potential vectors of *Salmonella* and *Erysipelothrix rhusiopathiae*, a bacterium that causes erysipelas. Erysipelas can cause mortality of up to 7% per day in hens as well as a 45% decrease in production and it has been detected in free range layer flocks (16).

#### Preventing contamination

The outdoor access increases the risks of contamination with viruses or bacteria carried by wild fauna. Avian influenza can result from direct contact with infected animals including wild birds, domestic animals or human beings, or indirect contact *via* water, the floor or buildings that have been contaminated by an infected individual. The presence of wild fauna was quantified in free range areas during a 12-month study in the Netherlands (17). A total of sixteen families of wild birds and five families of mammals were observed, but the results suggested that avian influenza virus was transmitted to poultry *via* indirect contact, i.e., contact with objects contaminated by wild fauna (17).

In order to limit direct and indirect contact with wild animals, biosecurity measures can be put in place to reduce the risk of infections, however these measures depend on the size of the farms. Avoiding puddles, concrete or pebbles around the house is the first step to prevent many contact risks. Nets is a common strategy to prevent high-risk birds from landing in the free range during times of avian influenza. Fences can be erected to limit contacts with ground animals and the attractiveness of the area for wild birds can be reduced with open landscape and by avoiding pools of water (18). However, open areas are used less than ranges with trees because of the anti-predatory behavior of hens. Therefore, some key informants mentioned that guard animals seem to be a good solution since they have a noticeable effect on preventing wild birds from landing and staying on the range (see 2.3 Risk of predation) (19). Nevertheless, the introduction of guard animals within the flock is forbidden for sanitary reasons by regulations in most European countries as these animals may themselves carry and transmit bacteria or viruses. A possible alternative is the use of lasers to repel wild animals (20). However, some recent outbreaks of avian influenza suggest that vaccination against the AI virus may be the most effective way to fight against this disease and changes in vaccination regulation could be considered.

Several strategies can be used to prevent contamination with parasites. In mammals, pasture rotation practices are used in order to limit infestation by endo-parasites. These practices are less common in poultry production because in most cases they would reduce the access of birds to the henhouse; however, this issue could be mitigated with the use of mobile housing systems, yet published demonstrations are lacking. The early detection of parasites can be facilitated by detection technologies that measure the parasitic load and enable farmers to intervene before the infestation level causes health and behavioral issues. *Ascaridia galli* is commonly detected using excreta egg count and serology, as antibody levels detected by ELISA in hen serum and yolk are correlated with infection intensity and the duration of exposure (21). As a consequence, antibody levels in hen yolk could be an early detection tool for *Ascaridia galli* infection. Reduced contamination by red mites can be achieved by the use of entomopathogenic fungi and predatory mites (15, 22, 23) that are very congruent with the requirements of organic production.

Systems that monitor microbiological water quality (24) can also help farmers to avoid contamination. Microbiological quality of the diet has to be taken in account, especially when the diet is prepared on the farm, since cereals or other raw material can be contaminated with bacteria or mycotoxins.

#### Strengthening immunity system functions

Infectious diseases can be fought by increasing innate immune defenses active on a large panel of infectious microorganisms *via* nutritional means or by using phytotherapy or aromatherapy (25). Nutrition was mentioned as an important point by key informants who were aware that it has widespread consequences on performances, but also health.

Nutritional requirements have to be fulfilled to ensure health and some of them are increased by the use of the outdoor range and the energy demand related to motor activity (26). Diet is usually considered to have a general effect on the immune system and has to prevent deficiencies. However, it can also improve resistance of laying hens to parasitic infection for example, when hens' diets are supplemented with omega-3 fatty acids (27). Among new diet components, insects and insect-derived products can provide a valuable amount of nutrients (proteins, lipids, vitamins, iron and zinc) while insect's antioxidant and antimicrobial peptides and chitin could stimulate the immune system and modulate gut microbiota (28). Diet can be used to provide nutrients that are involved directly in the defense mechanisms, but also to strengthen gut microbiota that will impact health, especially through the use of probiotic supplementation. Probiotics are micro-organisms which when administered in adequate amount confer a health benefit to the host. They are a single strain of bacteria or yeast or mixture of different strains and they can be included in animal food to improve the gut microbiota balance, and thus prevent or cure some health disorders. In organic hens, *Lactobacillus acidophilus* and *Bacillus subtilis* promote the presence of beneficial bacteria (probiotics) in the gut microbiota, while reducing the presence of potentially harmful bacteria (29). Many other lactic acid bacteria have also been shown to have probiotic activities in poultry (30) and *Lactobacillus rhamnosus* has been recently shown to exert a transient, beneficial effect on the immune response and tryptophan catabolism in pullets (31). Changes in gut microbiota can also be induced by prebiotics that are materials or nutrients that are used by bacteria and subsequently modify gut microbiota composition. Supplementation with prebiotics can also be used to stimulate immune responses and fight against some pathogens such as harmful bacteria but also endoparasites (32). Fermented diets can also improve gut health (33, 34). Nevertheless, an early adaptation to fermented diets during the rearing period seems to be necessary (35) and data about the use of such diets are still lacking in outdoor poultry production.

To limit veterinary drug use, preventing diseases by strengthening the immunity is part of an integrated management approach for animal health. For decades, herbal extracts have been used for their antioxidant, immunostimulatory, anti-inflammatory and antimicrobial properties in livestock (36–38). Plants, herbal extracts (phytotherapy) or essential oil (aromatherapy) containing bioactive compounds can be added to the diet, water or planted directly in the laying hens' habitat. Self-medication also appears as an interesting strategy. Chicks stressed by a delayed placement can also adjust essential oil consumption by uptake of lemon verbena essential oil known to have antioxidant, anti-inflammatory, sedative, and digestive effects (39) and it would be interesting to test this behavior in laying hens.

Use of phytotherapy requires adapted and reliable methodologies to select plants or their extracts and to evaluate the quality and the functional added value of the extracts for the health of the birds (40). For example, activity of *Melissa officinalis* on immunity and health has been demonstrated in a stepwise way (40) that consisted in testing *in vitro* activities, then *in vivo* testing with an inflammation model based on LPS injection and challenging more than 1,400 birds with sub-optimal conditions. This methodology can be used and adapted to the plant extract, the needs of professionals and staff responsible for health. Nevertheless, their effects on health are increasingly demonstrated and published, and the interviews confirmed that some veterinarians and farmers have used phytotherapy and aromatherapy for years.

The prophylactic use of vaccines is also possible but their use against bacteria and viruses varies among European countries; vaccination against *Salmonella* is not allowed in every country for example. Vaccines can be used to avoid coccidiosis since *Eimeria* parasites are highly immunogenic and many vaccine types are available; moreover, this prophylactic method is of high interest due to the increasing prevalence of *Eimeria* resistant strains to current chemicals. Since the authorized pesticides against red mites are now reduced, vaccination would be useful and one vaccine (Dg-CatD-1) seems to have a strong and long lasting efficacy in terms of considerably reducing the egg laying rate of the mites (41). The use of autogenous vaccines against infectious diseases is possible in free range laying hens (42), however it is not well-documented in the academic literature.

#### Curative methods

Phytotherapy and aromatherapy can also be used as curative since some of them have been demonstrated as toxic for pathogens (36, 38, 43, 44) and antifungal effects have also been described (38). Herbal extracts including essential oils can inhibit infectious agents such as bacteria (36, 37, 45, 46) or parasites such as coccidia (47–50). There are sometimes doubts about the effectiveness of alternative drugs against infection and the availability of literature is highly variable depending of the infectious agent. In the case of coccidiosis, a recent review (49) highlights the anticoccidial activity of several herbal products not in hens, but in broiler chickens. The activity of plant extracts against coccidiosis has been extensively studied and the ways plants fight against *Emeiria tenella* are various: some have general properties since they enhance the non-specific immunity, or show antioxidant, immune-stimulatory, anti-inflammatory properties, or maintain a healthy microflora and/or reduce secondary bacterial infection. They can also have specific activities toward *Eimeria* that decrease the fecal oocyst shedding, reduce the cecal lesions in infected chickens and lower the intensity of bloody diarrhea protecting infected broiler chickens from pathological symptoms. This description suggests that a combination of plants would increase efficacy and such studies are still needed. Such combinations have been tested in broiler chickens infected with sporulated oocysts of *Eimeria* spp. and this experiment demonstrated that one combination was effective in reducing lesions and oocysts output which was in line with the highest concentration of polyphenols (50).

The red mites have been commonly controlled with synthetic acaricides, but few products are licensed in the EU and many cases of resistance to these acaricides have been reported (51). Some alternative methods have been developed such as heat treatments in housing systems, but this method is not practical to use in wooden shelters. Silica-based products (Amorphous Si0_2_, diatomaceous earth) used as liquid preparation or diet supplementation with diatomaceous earth are currently used, but they can also be irritants for hens' skin and gut and humans and their effects may be variable (52). Some essential oils have repellent and toxic effects against red mites (43, 44). The effect on mortality was quantified after exposure to the essential oils on filter-papers and the repellent effects were assessed *via* mites' avoidance behavior toward the oils (43, 44). The combinations of plant extracts used on farm are based on the repellent and the toxic effects of the extracts, however their use has to be joined with usual biosecurity rules, and possibly heat treatment, to make protocols highly effective (53).

The proposed solutions against infectious diseases need further investigation to improve understanding of both economic impacts of infectious diseases and suitable inputs to use (range rotation, changes in nutrition, phytotherapy, aromatherapy, vaccination, etc.) in order to provide farmers with integrated advice.

### Non-infectious disorders

#### Bone lesions

**Figure 1:**
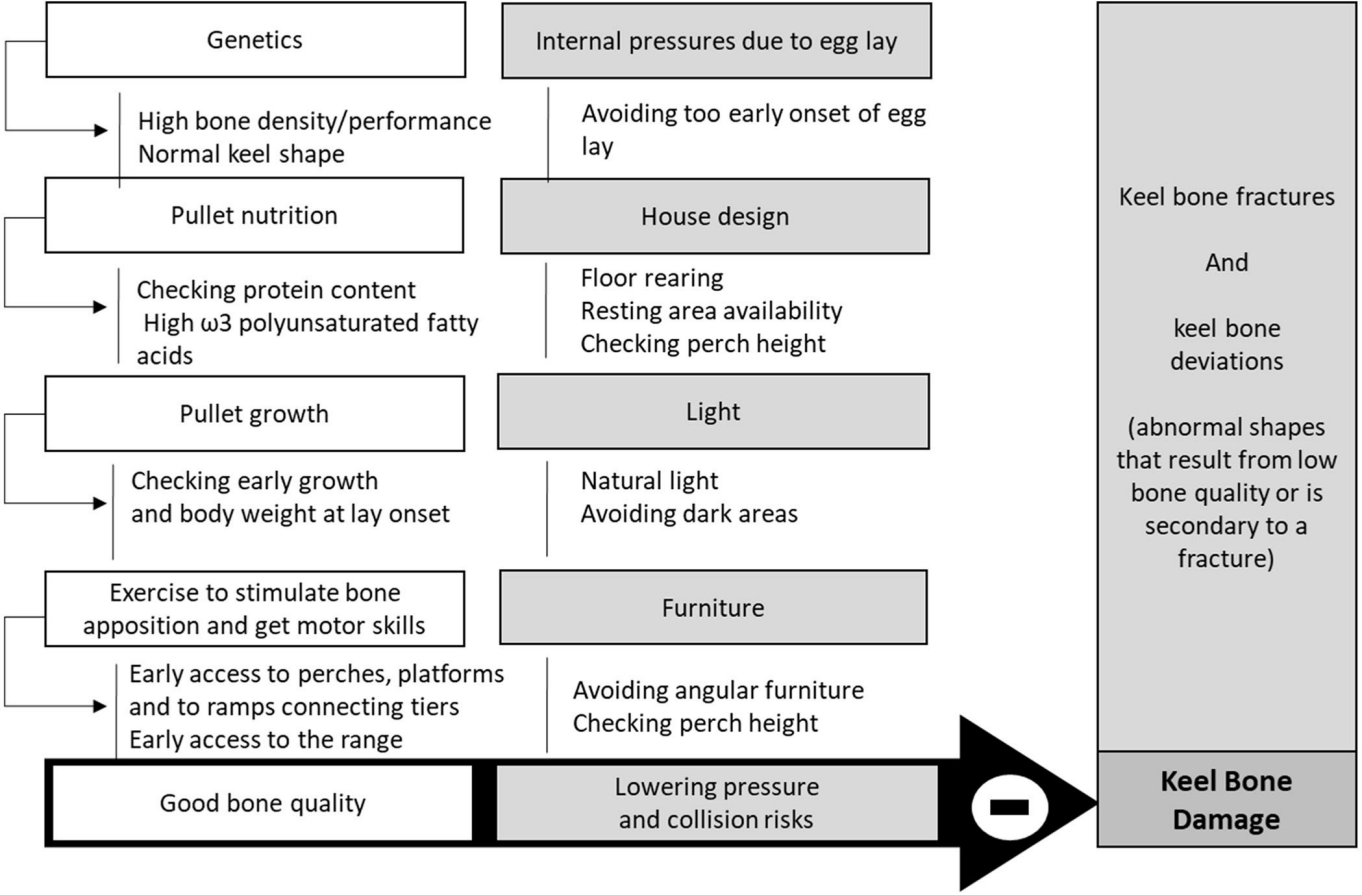
Practices to prevent fractures and keel bone damage [Overview from (55–60)].

Laying hens kept in free range and other cage-free systems can have high activity levels compared to caged hens and as activity in non-cage systems helps bone apposition (54), it is expected to reduce occurrence of skeletal problems. However, collisions with furniture in the house can lead to keel bone fractures, especially, but not exclusively, when bones are poorly mineralized. Moreover, as laying hens use a large amount of calcium for eggshell formation, this high use of minerals can encourage impaired mineralization of bone tissue and deviations of the keel bone (Figure 1) (61). These deformities could be primary or secondary to bone fracture (55) and the fractures have been mentioned as a serious issue by many key informants. Keel bone damage, including fractures and deviations, is a widespread welfare problem in both conventional and organic systems and its prevalence is expected to increase because the present or current trend is to lengthen the production cycle of laying hens (61). Keel damage prevalence ranged from 3 to 88% in a survey in organic flocks carried out by Jung et al. (56). Saraiva et al. (62) found that keel bone deviations were present in 60% of the hens reared in free range systems while Bestman et al. (63) reported that they were present in every flock affecting on average 21% of the birds. However, it appears that the prevalence of fractures and deviations are underestimated by the commonly used palpation method (55).

According to the trauma hypothesis proposed by Wilkins et al. (57), fractures would result from collisions with the perches, platforms or other objects in the environment. The housing system and its design thus influence both bone strength and the incidence of bone fractures (Figure 1). According to Jung et al. (56), the main risk factors are aviary vs. floor systems and the absence of natural daylight in the hen house, linked with increased collision risks. In this study, a higher percentage of underweight hens and a higher laying performance were found to be associated with a higher prevalence of keel bone damage, which can be explained by poor bone quality related to underweight birds or calcium exportation. In Thøfner's study, the accurate examination of keel bones demonstrated that most of the fractures were in the caudal parts of the keel and could not be explained by trauma (55). They were more likely explained by poorly mineralized bone tissue in the pullets, especially with low body weight, and the high demand for calcium for eggshell at a point when bone growth is not yet completed. Pullet rearing methods and genetic selection for early lay and large eggs thus also have an impact on bone damage in hens, particularly due to the late ossification of the full keel bone (58).

The addition of omega-3 polyunsaturated fatty acids has been mentioned to improve bone strength and reduce bone breakage (64). However, the inclusion of such a high percentage of these fatty acids in the ration needs to be assessed for its economic viability as its addition would be costly. Other diet improvements are being tested, especially with probiotics (65), but the benefits remain to be proven. Here again, improving diet can be an effective way to improve health, which explains that feeding is considered as a pivotal topic by the key informants.

#### Thermal stress

**Figure 2:**
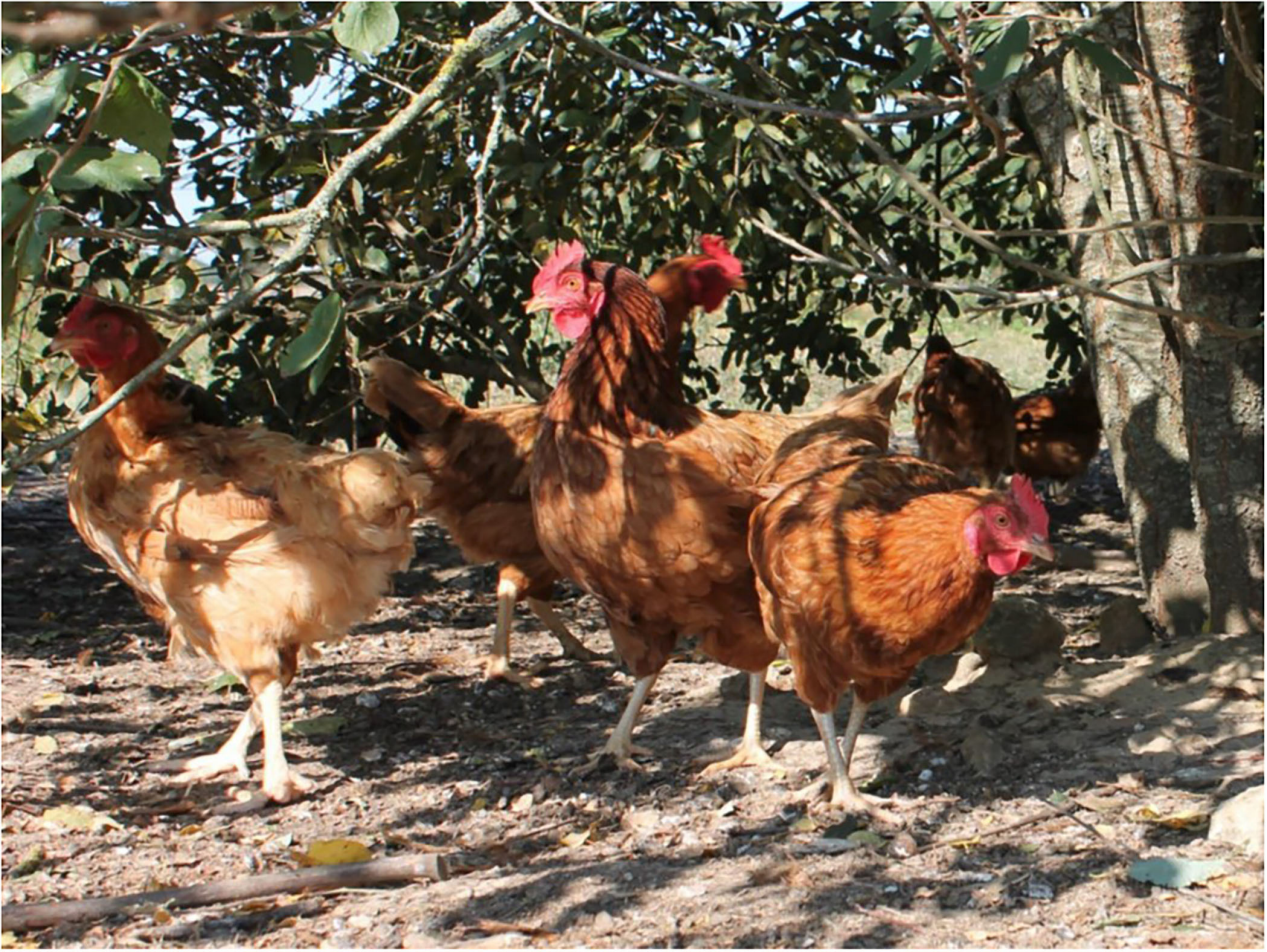
Hens under bushes creating a fresh microclimate.

Animals housed in free range systems are subject to variable weather conditions and coping with such conditions was mentioned as a difficult issue by several key informants. However, whether free range hens are truly more likely to suffer from thermal stress still needs to be substantiated by scientific evidence. At least in theory, free range hens may have more means for dealing with thermal stress by seeking places in the free range with shelter or a better microclimate, or they can choose to go inside when the weather is aversive. Heat stress decreases feed intake and has a general negative impact on behavior, performance (egg production, egg quality), growth and health (66). Some crossbreeds used in free range production are more resilient to heat stress than pure breeds (67). When there is no shadow available on the range, hens stay indoors to avoid the sunshine (68). This behavior increases the stocking density indoors and hens may suffer from heat stress since housing used for free range production is sometimes not as well-ventilated as that used for conventional production systems. Thus, providing shadow on the range helps hens limit their heat stress since they are less exposed to sunshine (69, 70) and can dig holes to keep cooler through contact with the cooler ground (Figure 2). Covered verandas can also provide shade and a transition from the house to the free range and they can also be useful during cold weather to avoid cold coming in through the pop holes. Cold, rain and wind can limit range use and many key informants mentioned weather conditions are an issue that is difficult to cope with. The location of the henhouse is thus pivotal in alternative systems and it should be situated in areas protected from strong winds, surrounding fields should be effectively drained and the house should have good thermal insulation properties.

### The risk of predation

As mentioned earlier and by several key informants, poultry going outside are more exposed to predators, whether it is from ground-based predators or from the air by birds of prey or corvids. Ground-based predators, such as foxes, dogs and mustelids can be kept out by a good electric fence around the free range. Protecting from aerial predators is more difficult. In the Netherlands, animal losses due to predation were evaluated at ~3.7% by 61 farmers who responded to an online survey, while an on-farm experiment showed that out of 44 hens killed by predators, 32 deaths were due to birds of prey and dour due to foxes (71). A similar survey concerning fox predation in the UK completed by 58 egg producers (72) showed that <2% of hen mortality was due to fox predation. In all these cases, mortality may have been underestimated since farmers do not record all cases of predation.

Fences protecting for ground predation and guard animals against crows and raptors were considered by some interviewees as the best methods to protect laying hens from predators compared to scaring devices since predators can get used to them. A recent study of guard dogs by Zingaro et al. (73) found that they stay with the flock even when unsupervised. Similarly, the Hennovation project found that Alpacas are also effective guard animals as they are social but territorial (74) and some informants also mentioned donkeys as guard animals. However, the use of guard animals in poultry production is still relatively rare and dogs' behavior toward predators still needs investigation (19). The use of netting is more and more widespread, even if nets above wide range areas are difficult to manage and expensive. Indeed, nets avoid contact with wild birds that could carry avian influenza virus. Automated laser devices could be new tools to repel wild birds since they reduced the birds visiting the range by 98% (20).

## Behavior issues in laying hens in organic and free range systems

The provision of an outdoor area provides opportunities for the expression of many behaviors such as exploratory behavior, comfort behavior (resting, dust and sun bathing, wing stretching), feeding behavior (catching snails, insects, eating leaves), running, playing, etc. Moreover, hens using the outdoor area have less plumage damage, indicating less feather pecking (75) and the use of the range has implications on some health variables (8). The use of the range raises many questions since it may not be used enough if it is not attractive enough for the hens or it may get damaged by crowding and intensive use if it is not designed to facilitate the use of the whole range area (76). As a consequence, range use and range management are issues that were mentioned by key informants in each country. Feather pecking was also mentioned as a behavior issue by key informants.

### The use of the outdoor area

The percentage of hens seen on the range at any time varies between 0% under bad weather conditions to different percentages according flocks and studies. For example, the mean percentage of hens outside in non-organic flocks studied in the Netherlands was 23% and the variation coefficient of this percentage was 65% while the mean percentage in Switzerland was 48% with a variation coefficient around 40% (77). Indeed, studies have shown that the use of the range by laying hens is limited and mainly influenced by weather and the design of the range (78). Nevertheless, other studies have shown that the percentage of hens frequently using the pop holes is above 80% (79–81). The variations in the use of the outdoor area between farms can be explained by environmental reasons including differences such as weather conditions as mentioned above, range design and stocking density, while intra-flock differences appear to be related to personality and experience of the hens.

#### Range vegetation and enrichment

Many hens stay close to the house and do not use the whole range when trees, shrubs or shelters are not available (77) and this behavior often leads to damaged areas close to the pop-holes. This lack of use of some ranges such as open pasture can be partly explained by the anti-predatory behavior of the hens and it appears that providing continuity between the house and the range through plants and line-shaped elements helps to increase the distance walked from the house. The addition of a shelterbelt composed of trees of different heights (1, 2, and 3 m) at 10 and 20 m from the henhouse almost doubled the percentage of hens observed at more than 20 m from the house (82). According to Nagle and Glatz (82), the addition of a shade cloth at 10 and 20 m from the shed led to almost twice as many laying hens being observed outside in the morning. Enrichment, such as bales of alfalfa, can be used in outdoor production systems just as they are used in conventional indoor systems (83). However, in the former context they can also act as a tool to enhance the use of the range. The use of drinking and feeding points outside is limited by rules against avian influenza virus, but feeding motivation can be used to increase range use by planting attractive plants (chicory, *Artemisia annua, Aronia melanocarpa*, etc.) (84) or spreading insect larvae such as black soldier fly larvae or meal worms (85). Maintaining a dry area to promote dustbathing in the range can also help range use since this behavior is commonly seen outside.

#### Flock size and stocking density

The effects of flock size and stocking density can hardly be separated in practice on farm since low density is often associated with small flock size and key informants frequently linked both parameters. Chielo et al. (86) found that the percentage of the flock out of the shed increased from 6.3 to 35.1% as the size of the flock was reduced from around 15,600–3,900 hens. Similarly, Bestman et al. (77) showed that for flocks composed of around 24,000 or 8,600 individuals, 23 and 52% of the hens went outside, respectively.

Data show that outdoor density has some effects on the range use in small flocks. In small experimental flocks of 150 laying hens housed with access to ranges with low, medium and high outdoor stocking densities (0.5, 1, or 5 m^2^/hen), hens from the lowest stocking density on average used the range for longer each day by weeks 32–36. Moreover, the proportion of hens that accessed the range daily was 80.5, 66.5, and 71.4% in the flocks with low, medium, high stocking density, respectively (87). In another experimental design, organic laying hens from small flocks (around 400, 600, and 800 hens) were housed in a multi-tier system with permanent access to an outdoor area with a veranda and were kept at inside stocking densities of 6, 9, and 12 hens/m^2^ available floor area. Hens kept at the lowest stocking density outside and the smallest group size appeared to use the outdoor area more extensively (88). However, studies on the effects of densities in larger flocks with comparable sizes are missing.

#### Early rearing influences

Range use is influenced by external factors as previously mentioned and by factors related to the hens. Early experiences of enrichment have been found to enhance range use. Laying hens provided with various enrichments (wallpaper, novel objects attached to feeders and water nipples, flashing lights, auditory recordings, moving vehicles, etc.) visited the range more often than birds lacking such enrichments across the first 4-weeks of range access (89). Additionally, pullets provided with enrichments showed longer maximum visit times than control hens when aged between 25 and 64 weeks old (90). Thus, early life experiences influence later range use.

#### Personality and range use

Some hens never go outside even when the environmental conditions are favorable and this seems to be linked to individual personality. Previous studies mentioned personality traits related to foraging behavior (91) and ranging patterns (92). Using radio-frequency identification (RFID) technology, Campbell et al. (92, 93) were able to use hens' ranging behavior to classify them as indoor hens that rarely went outside, and outdoor hens that accessed the range daily. Another study showed that hens aged 20–36 weeks that never used the range were less likely to use it later, while hens that used the range intensively over the same life interval were more likely to use it later on (75). Whether low range use is related to higher fearfulness is still under investigation. According to Hartcher et al. (79), low range users whose total duration outside was 16.7 h over 13 days, had around 50% longer tonic immobility durations than high range users whose total duration outside was 142.5 h over the same period, suggesting a higher fearfulness in low ranger hens. However, this relationship between tonic immobility duration and range use has not been established in other studies (94, 95). Fearfulness measured in an open-field test and in an emergence test was also higher in low ranger hens (92, 94).

Hence, it appears that more research is required to understand better how the environmental characteristics of the range impact exploratory behavior and which personality traits are related to the propensity of each hen to forage on the range (96).

### Feather pecking

**Figure 3:**
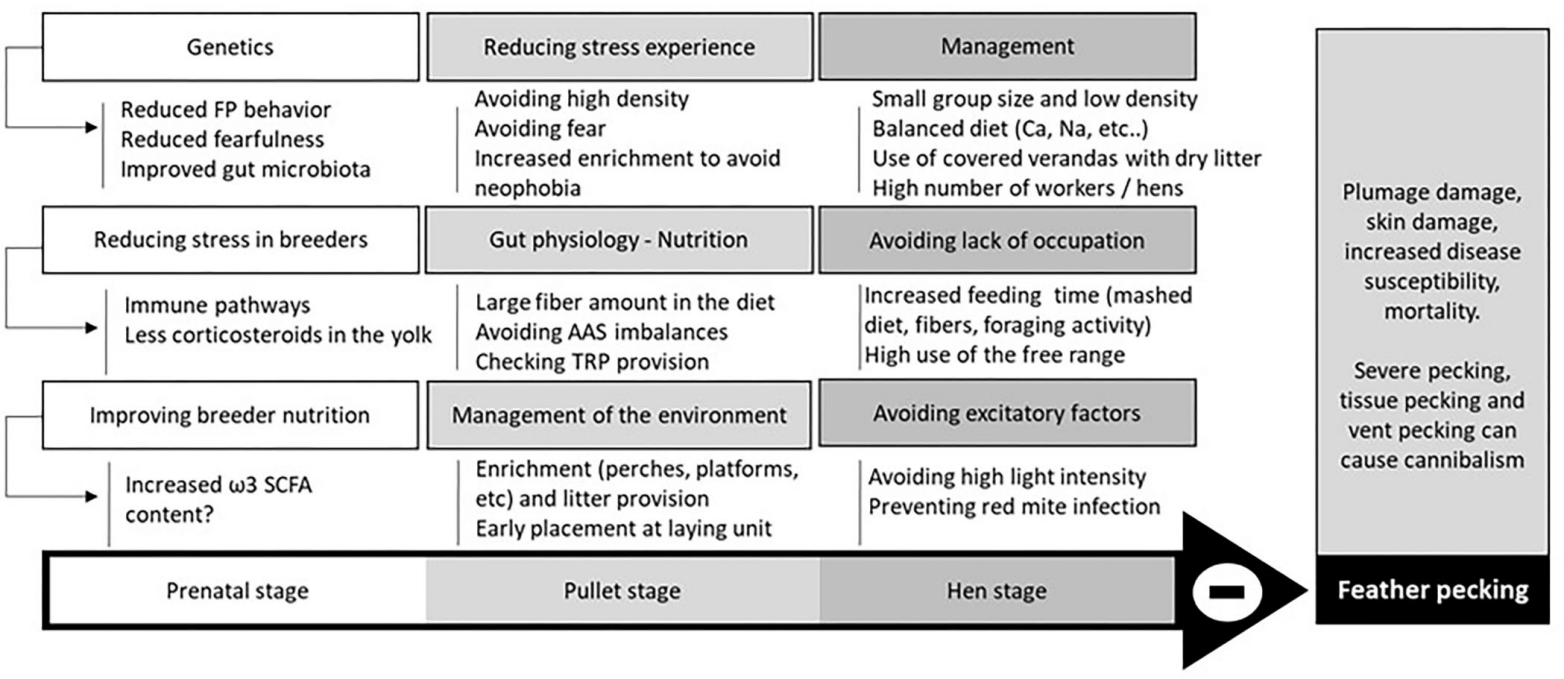
Practices to prevent feather pecking [Overview from (97–103)]. AAS, Sulfur amino-acids; FP, Feather pecking, SCFA, short chain fatty acids, the increase in omega-3 SCFA in breeders' diet decreases FP in the offspring in ducks (98), but this demonstration is lacking in hens. TRP, Tryptophan, this molecule can influence FP through serotonin synthesis and the gut microbiota composition.

Feather pecking is a behavior that is influenced by many environmental factors and its occurrence can thus vary greatly under free range conditions. Feather pecking extends from gentle pecking that is considered a normal social behavior, through to severe pecking that can induce pain in the victim, or in extreme cases can even leave birds featherless and lead to cannibalism (Figure 3). There are multiple factors leading to severe feather pecking (SFP) in laying hens and there are multiple hypotheses as to why SFP outbreaks start. The redirected foraging hypothesis assumes that foraging behavior is a natural behavior of hens and if deprived of opportunities to forage, hens will start to peck at each other, potentially creating a SFP episode (91). However, the redirected foraging hypothesis has been somewhat revised by Newberry et al. (97) who concluded that, although indeed birds that show high levels of ground pecking when young are more at risk of developing feather pecking, the latter does not replace ground pecking. In their study, adult feather peckers continued to show high levels of ground pecking as well. Whatever the pathogenesis of SFP, prenatal and post-natal factors influencing this behavior are numerous (Figure 3) and some free range conditions can offer solutions to prevent it.

#### Influence of genetic and prenatal factors

Several studies (104–111) have demonstrated that genetics influence the risk of feather pecking and that it is possible to reduce feather pecking by genetic selection, as the heritability of gentle and severe feather pecking is sufficient for selection (112). Indeed, Brinker et al. (113) showed that breeding for improved plumage condition can be strongly enhanced by using the recently developed indirect genetic effects models, in which the genetic influence of an animal on the plumage condition of its group members can be estimated (111). The main issue in genetic solutions for free range production is that selection against feather pecking is not carried out under free range conditions, which may lead to reduced effectiveness of this selection. Dual-purpose hybrids can be used to limit feather pecking (114, 115) and they also provide a solution to avoid the elimination of 1-day old chicks. Moreover, some dual-purpose hybrids are less fearful than conventional layer hybrids which might make them less sensitive to stress during management routines (116).

The experience of breeders and their sensitivity to stress can also influence severe feather pecking behavior in their offspring. De Haas et al. (117, 118) showed that one of the major risk factors for feather pecking to develop in commercial rearing flocks was stress in the parent stock, as evidenced by high activation of the hypothalamus–pituitary–adrenal (HPA) axis and increased feather damage. This was mainly the case in flocks from a White Leghorn genetic background. Nutritional factors in breeders can also impact feather pecking. This has been demonstrated in ducks with a diet enriched with omega 3 short chain fatty acids (98) but has not been established in hens yet.

During the incubation period, exposure to light can influence feather pecking in those birds once hatched. Riedstra et al. (119) were the first to report that light (type and duration) during incubation had an effect. They found that exposure to white light 3 days before hatching led to an increase in gentle feather pecking in the chicks. More recently, Ozkan's group studied the effects of 16 h of light per day during incubation in broilers (120) and laying hens. In laying hens, they found that compared to incubation in the dark, white light increased feather pecking, while exposure to green light would reduce it post-hatching (121).

#### Impacts at the pullet stage

According to Bestman et al. (122), 71% of pullets that did not engage in feather pecking at the pullet stage would not do so at the laying stage either. However, at the pullet stage, it is possible to observe severe feather pecking and feather pecking that develops during the rearing period increases risks in the laying period (123). Selection of low and high feather pecking laying hens strains has been positively linked to locomotor activities and feather pecking (123). However, selection of high and low general locomotor activity did not confirm this result (124).

An enriched environment, for example providing pullets with litter, will help prevent feather pecking during the laying period (58) and the absence of litter during early life is a major risk factor for feather pecking to develop (117, 118). Providing pullets with a rewarding enrichment, such as hay, reduced the number of aggressive pecks at 27 weeks compared to hens that were not enriched during their early life (125), while enrichment with plastic boxes was not effective. It also seems worthwhile habituating both parent stock and the rearing flock to humans and human activities, to make the birds less sensitive to human disturbance, since fear of humans is another major risk factor for this behavior (117, 118). A recent review by Mens et al. (102) concluded that the effects of pullet nutrition on feather pecking are based on two main routes. The first one uses the effects of nutrients that act on physiological mechanisms that avoid deficiencies and imbalances (dietary protein, amino acids) or on gut microbiome (tryptophan for example, used in serotonin synthesis, a neurotransmitter involved in feather pecking). The second route is based on the nutrition effects on feeding behavior and satiety. This strategy aims at increasing feeding time with fibers in the diet and occupation with foraging stimulations.

Finally, access to the range has an influence on the type of pecking shown (gentle, severe or aggressive) and on the total number of pecks. In fact, early access to the range at 18 weeks rather than 22 weeks, resulted in a reduction of pecking behavior (126). This may be related to a better habituation to the range and because the stress induced by this new environment does not overlap the numerous physiological changes experienced at the onset of laying.

#### Strategies to limit feather pecking during the laying period

Several studies have demonstrated a relationship between range use and feather pecking among laying hens kept in indoor and free range systems (127, 128). A survey of 1,000 flocks by Bright et al. (129) demonstrated that less canopy cover within tree-planted areas resulted in poorer plumage condition at the end-of-lay and they suggested that the degree of shade encourages the hens outdoors to range, thereby reducing feather pecking. According to Bestman and Wagenaar (130), the presence of cockerels within the laying hen flock encourages the laying hens to make a better use of the range which, in turn, leads to reduced feather pecking. However, this factor was not confirmed by Jung and Knierim (101) in their epidemiological study. The use of foraging material is mandatory in organic poultry production (EU Council regulation 1804/1999) and daily access to this seems to have positive effects on behavior as it motivates hens to spend time foraging, which can reduce the incidence of feather pecking and mortality (131, 132).

Nutrition also affects feather pecking through diet composition that helps avoid deficiencies and imbalances, and through presenting feed in a way that favors the time spent to feed (103, 133). Key informants were aware of the impact of feeding on this behavior and many of them mentioned feeding as an issue. Among others, the provision of sulfur amino acids and tryptophan natural sources should be investigated carefully because sulfur amino acids are found in feather composition and because tryptophan influences serotonin synthesis. Serotonin metabolism appears to be altered during severe feather pecking (134). Fiber content is also pivotal for feather pecking to develop since a high fiber diet increases the time spent feeding, and also because the fiber content influences gut microbiota composition whose imbalance can also be related to feather pecking. An interesting option for feeding enrichment to reduce feather pecking could be to supply insect larvae. Black soldier fly larvae and house fly products have been examined as an alternative to soy in diets and it appears that they have the potential to improve feather coverage as well as providing the same levels of performance and egg quality as soy products (135). Furthermore, black soldier fly larvae might reduce feather pecking in laying hens (136), a characteristic that has already been identified in turkeys (135). Supplying insect larvae to laying hens thus seems a very natural way to stimulate normal exploratory and foraging behavior and to reduce the risk of feather pecking especially in organic production where the use of synthetic amino acids is forbidden (137).

It appears then that many of prevention strategies are facilitated in free range production systems even if the influence of genetics, prenatal and nutritional factors require further investigations in free range hens.

## Conclusion

This article reviewed the scientific data regarding specific welfare issues that are encountered in free range and organic laying hen systems and the selection of the main issues was strengthened by the interviews of key informants. Many different measures have been explored to counterbalance them. While these systems allow the expression of a wide range of behaviors, exposure to some diseases, adverse weather and predation is increased by the outdoor living conditions. Nevertheless, a wide array of solutions exists to combat these collateral effects, through the use of different genotypes and management strategies (nutritional strategies, design and management of the range, etc.). Among these solutions, it is important to highlight that adapting early rearing of pullets is a pivotal phase to help improve hens' life quality. The management of the range is a key point to reduce health issues, especially predation and heat stress, and to increase range use and prevent severe feather pecking. However, further knowledge is still required about the ways to enhance the expression of natural behaviors and the role of hen personality, the efficiency of alternative drugs against infectious diseases and the influence of early life conditions.

This review provides information about practices that have been tested or still need to be explored and this overview of the literature and expertise of key-informants can be used by stakeholders and researchers to help them evaluate the applicability of these solutions for welfare improvement.

## Author contributions

Published literature and research projects' results were collected and reviewed by CB, AC, LG, VG, SR-G, TR, FT, SS, LB, JN, and CL. The interviews were prepared and carried out by CB, LW, MR, RP, AZ, PV, PP, KW, MV, and CL. CB, AC, LG, VG, SR-G, TR, FT, SS, LB, JN, and CL wrote the manuscript. All the authors reviewed and approved the final manuscript.

## Funding

The PPILOW project has received funding from the European Union's Horizon 2020 research and innovation program under grant agreement N°816172.

## Conflict of interest

The authors declare that the research was conducted in the absence of any commercial or financial relationships that could be construed as a potential conflict of interest.

## Publisher's note

All claims expressed in this article are solely those of the authors and do not necessarily represent those of their affiliated organizations, or those of the publisher, the editors and the reviewers. Any product that may be evaluated in this article, or claim that may be made by its manufacturer, is not guaranteed or endorsed by the publisher.

## Supplementary material

Supplementary Data S1 Detailed characteristics of alternative systems for laying hens.

Supplementary Data S2 Methodology and main results from interviews with key informants.

Supplementary Data S3 ATOL definitions.
