# Supplementary material for: Welfare issues and potential solutions for laying hens in free range and organic production systems: A review based on literature and interviews
Source: Front Vet Sci. 2022 Aug 5;9:952922. doi: 10.3389/fvets.2022.952922 (PMC9390482; doi:10.3389/fvets.2022.952922)
Supplement: Supplementary Data S1 — Detailed characteristics of alternative systems for laying hens. [file Data_Sheet_1.PDF]

Supplementary data 1

Characteristics of laying hen production systems

The specifications of (EU) 2018/848 and implementing regulation (EU) 2020/464 determine the characteristics of organic laying hen production. The EU organic regulation pays attention to e.g. flock size, range characteristics, space allowance, bids, housing, enrichments and bird nutrition. There were also requirements on the number of roosters in the flock.

The nests to lay eggs and the use of these nests are essential characteristics in laying hen houses. Organic systems paid additional attention to the size and number of nests or nesting area. Lighting was also emphasized in poultry systems. This included natural lighting, which can be complemented with appropriate artificial lighting periods, and an adequate rest period at night. Another specific feature in organic laying hen systems was perches. Perches were required in organic laying hen houses, but some voluntary systems had an additional lever and provided additional perch space to the birds. Aerial perches were said to allow birds to exhibit a greater range of natural behaviours, and enabling birds to escape from any ground level harassment from other birds. Mutilations were often prohibited in organic laying hen systems. Some systems allowed a molting or a laying break under special conditions to enhance the renewal of feather coverage thereafter. However, artificial molting induction is normally forbidden.

Directives 1999/74/EC and EC/589/2008 specify the typology of laying hen production in the EU. Besides conventional and organic production, there are other ‘alternative’ production. If hens have outdoors access e.g. to appropriate winter garden, but not to free-range, and the requirements of alternative production are met, these eggs are called ‘Barn eggs’. Eggs can be called free-range eggs only if they meet also the EC/589/2008 definition of free-range.

The levers mentioned for alternative (low-input) systems were related especially to outdoor access, space allowance, environmental enrichments, mutilations and feed (see Annex 9). Access to winter garden was required for some systems, and usually these were barn systems. Specification of the size and furniture of the winter garden however varied. The outdoor range (other outdoor area than winter garden) was typically required to be permanent grassland and contain trees (i.e. an agroforestry system) or other shelters. Shelters or vegetation provide protection from adverse weather and predators. The space allowance on the range varied from below to above the requirement of organic production. However, overall, low input systems tended to position their space allowance and nest requirements along the lines of various directives, including especially the directive on organic production even if the production was not organic. Resources to facilitate the scratching of the ground, dust bathing, foraging and other natural behaviors were also used as levers to enhance animal welfare.

Sufficient daylight, good climatic conditions in the housing as well as low dust exposure were mentioned as preconditions for the health and welfare of poultry. Both the winter garden, if it exists, and the housing could be illuminated. Besides satisfying the needs of the hen, the lighting regime shall prevent health and behavioral problems and meet the physiological requirement of the hen in order that they lay well all along the year. The 24-hour rhythm shall include an adequate uninterrupted period of darkness lasting (one third of the day) to allow rest and to avoid welfare problems. Dimming the lights so that the hens may settle down without disturbance or injury was also required explicitly in some cases.

Litter management was highlighted strongly by all poultry production systems as a key factor of production, and the quality of litter was emphasized. High level of biosecurity was required by all the systems, but this is mostly a legal requirement too. Especially outside areas may facilitate contact with wild birds and thereby increase the risk of parasitism or risks of contacts with Influenza virus or other microbes carried by the wild birds. The levers mentioned by the systems also paid attention to reducing the mortality, which tends to be higher in floor rearing systems than in enriched cage systems, and to the duration of laying period.

Supply chain structure

Organic production systems in the EU are certified by definition. While some low-input systems were certified by an external party not involved in the production chain, other low-input systems were not certified. While there were several low-input systems which represented either a short supply chain or were based on local production and animal welfare was just one of the features profiling the system, there were also low-input systems which represented a conventional supply chain. In addition, some systems were officially recognized (e.g. Protected designation of origin (PDO)) which links the rearing to specific geographic, climatic and environmental conditions. Highlighting local production appeared to be an important economic lever. These characteristics were important in low-input systems of both pigs and poultry.

Mandatory requirements in organic laying hen production systems (standard EU organic requirements and features promoted by various voluntary organic schemes in addition to the EU regulation).

**Legend**	**EU organic ((EU) 2018/848, (EU) 2020/464)** https://eur-lex.europa.eu/legal-content/EN/TXT/PDF/?uri=CELEX:32018R0848&from=EN https://eur-lex.europa.eu/eli/reg_impl/2020/464/oj
Animals, reproduction and slaughter	Account for the capacity of animals to adapt to local conditions, their vitality and resistance to disease. Prefer indigenous breeds and strains. Use organic animals (with some exceptions). Either a minimum age of 81 d or a slow-growing breed
Confinement	No cages allowed.
Housing	Housing not mandatory.<br>≤3 000 laying hens per compartment.
Space allowance, indoor	Sufficient space for birds to stand naturally, lie down easily, turn round, groom, assume all natural postures and make all natural moves such as stretching and wing flapping, account for the behavioral needs. A sufficient part of the floor area available to the hens for the collection of bird droppings. Net indoor area ≤6 laying hen/m^2. ≤7 laying hens/nest or in case common nests, ≥120 cm^2/bird.
Space allowance, outdoor areas	Outdoor area available in rotation is ≥4 m^2/bird provided that the limit of 170 kg of N/ha/year is not exceeded
Floor characteristics	≥1/3 of the floor area solid and covered with a litter material (e.g. straw, wood shavings, sand).
Light	Plentiful natural light to enter, ≤16 hours/day. Continuous nocturnal rest period ≥8 hours/day.
Ventilation and air	Natural ventilation. Air circulation, dust level, temperature, relative air humidity and gas concentration that is not harmful to the birds.
Outdoor access	Easy access to open air area. Exit/entry pop-holes ≥4 m per 100 m^2 area of the housing.<br>When confined indoors due to community legislation, sufficient quantities of roughage and suitable material to meet ethological needs is required.
Type of outdoor area	Open or partly covered.<br>Open air areas mainly covered with vegetation and providing with protective facilities and adequate numbers of drinking and feeding troughs.
Environmental enrichments	Perches required.<br>Perch length available to animals: ≥18 cm/laying hen.
Veterinary policy	Chemically synthesized allopathic veterinary medicinal products or antibiotics for preventive use, growth promoters, hormones or similar substances to control reproduction or for other purposes is prohibited. Phytotherapeutic, homoepathic products, trace elements and products listed in the directive are preferred, if effective. Vaccinations, parasitic treatments and compulsory eradication schemes allowed.
Allowed mutilations	No *routinely* operations such as trimming of beaks.
Feed-related aspects	Organic feed. Roughage, fresh or dried fodder, or silage shall be added to the daily ration for poultry. Force feeding prohibited.
Other management	Housing, equipment and utensils cleaned and disinfected to prevent cross-infection and the buildup of disease carrying organisms. Organic substances only. Faeces, urine, uneaten feed removed to minimise smell and to combat insects or rodents. Cleaning and disinfection of facilities between flocks, production break to allow vegetation to grow (specified by MS).
Certification	By competent authority.
Supply chain characteristics	National organic associations typically involved in the value chains.
Other remarks	Stocking density on the farm ≤170 kg of nitrogen per year and hectare of agricultural area. Landless livestock production prohibited.

Mandatory requirements in alternative (1999/74/EC, EC/589/2008) low-input non-organic outdoor laying hen production systems.

**Legend**	**Mandatory requirements in alternative non-organic systems**
Animals	
Confinement	No cages allowed in the alternative systems.
Housing	The floors of installations to support adequately each of the forward-facing claws of each foot. If there are levels and hens can move freely between different levels, then the number

	of levels is ≤4, the headroom between the levels ≥45 cm, hens have equal access to drink and feed, and the levels are arranged to prevent droppings falling on the levels below.
Alternative patterns of outdoor access	Not required in barn production. If hens have outdoors access e.g. appropriate winter garden, but not to free-range, and alternative production requirements (1999/74/EC) are met, these eggs are called 'Barn eggs'. *The eggs can be called free-range only if they meet also the free-range definition of EC/589/2008*<br>In all alternative systems, if laying hens have access to open runs, several pop-holes are required, which ≥35 cm high and ≥40 cm wide, and extending along the entire length of the building. A total opening of ≥2 m per group of 1 000 hens. Open runs have an appropriate stocking density and ground, to prevent any contamination, and equipped with shelter from inclement weather and predators and, if necessary, appropriate drinking troughs.<br>*Free-range systems: continuous daytime access to open-air runs, which can include a limited time period of non-access in the morning hours. Open-air runs must not extend beyond 150 m from the nearest pophole. Exception: 350 m is allowed if there are sufficient shelters (≥4shelters/ ha) evenly distributed in the open-air run.*
Type and management of outdoor area	*Free-range systems: Open-air runs must be mainly covered with vegetation and not be used for other purposes except for orchards, woodland and livestock grazing.*
Space allowance	The stocking density is ≤9 laying hens/m^2 usable area. ≥250 cm^2 littered area/hen and the litter occupying ≥1/3 of the ground surface.<br>There is at least one nest for every seven hens. If group nests are used instead of individual nests, the nest space is ≥1 m^2/ 120 hens<br>*Free-range systems: open-air run space ≥4 m^2/hen at all times. Exception: If ≥10 m^2/hen is available, rotation is practised and hens have even access to the whole area over the flock's life, each paddock used must at any time assure ≥2,5 m^2/hen;*
Light	Light sufficient to allow all hens to see one another and be seen clearly, to investigate their surroundings and to show normal levels of activity. If natural light, light apertures to be distributed evenly within the accommodation. The lighting regime shall prevent health and behavioral problems, and the 24-hour rhythm shall include an adequate uninterrupted period of darkness (one third of the day) to allow rest and to avoid problems such as immunodepression and ocular anomalies. The dimming of lights so that the hens can have time tosettle down without disturbance or injury.
Environmental enrichments	Perches without sharp edges, ≥15 cm/hen, not mounted above the litter and the horizontal distance between perches ≥30 cm and the distance between the perch and the wall ≥20 cm are required. ≥250 cm^2 littered area/hen, ≥1/3 of the surface.
Veterinary policy	
Allowed mutilations	All mutilation prohibited (98/58/EC). However, to prevent feather pecking and cannibalism, several MS have authorize beak trimming when made by qualified staff on chickens ≤10 days old and intended for laying.
Feed-related aspects	In alternative systems, all laying hens have either linear feeders ≥10 cm/bird or circular ≥4 cm/bird; continuous drinking troughs ≥2,5 cm/hen or circular drinking troughs 1 cm/hen (where nipple drinkers or cups are used, ≥1 nipple drinker or cup for every 10 hens, and where drinking points are plumbed in, ≥2 cups or nipple drinkers within reach of each hen);
Other aspects	All hens inspected at least once a day. Minimized sound level.<br>Buildings, equipment or utensils in contact with the hens cleansed and disinfected regularly, and every time a depopulation is carried out and new hens are brought in.<br>Droppings must be removed as often as necessary and dead hens daily.
Certification	Not required.
Supply chain characteristics	
Other remarks	

Supplementary data 2

Interviews of key informants (Guidelines and results related to welfare issues)

The interviews took place within the PPILOW project aiming at improving the welfare of pigs and poultry low-input outdoor and organic production systems, however only data related to welfare issues in laying hens were used in this review. An information form was sent to the key informants several days before the interviews and a consent form was signed by the interviewees before the interview starts. Both forms and the guidelines were approved by the French ethics committee Polethis from Paris-Saclay university.

A- Interview guidelines

Italics indicate the sentences used for non-farmers when the questions have to be changed between farmers and non-farmers.

**Topics**	Mandatory questions	Optional questions which could reactivate the discussion
**Opening question**	Could you please describe animal welfare according to you? How do you measure/assess it?	
**Topic 0 :** Farming systems and their definition (5 minutes)	- By which criteria would you define low-input outdoor or organic poultry production?<br>- Which different low-input outdoor or organic poultry farming systems are you aware of in your country?	- How do different outdoor or low-input organic production systems differ?
**Topic 1** : Perceptions on animal welfare (30 minutes)	- How do you define animal welfare?<br>- According to you, how did animal welfare evolve and what has boosted this evolution?<br>- In general, which are the main challenges and concerns related to (species the farmer is dealing with)'s welfare in organic and low-input outdoor farms in (country of the interviewee)?<br>- In general, which farming practices do you think **reduce** animal's welfare in outdoor or low-input organic production systems? In general, which farming practices do you think **improve** animal's welfare in outdoor or low-input organic production systems?<br>- In general, which farming practices are promoted but do not seem to have an impact on animal welfare?<br>- How can <u>you</u> improve the welfare of animal reared by you? Which measures (levers) would you use, or expect other actors to use, in order to promote the adoption of animal-friendly farming practices?	- How would you measure animal welfare? How did you acquire knowledge about animal welfare (experience, courses…)?<br>- How do you think those challenges emerged? Could it be due to laws and regulations? Or voluntary applied farming practices? Or changes in the farming systems or the farming environment?<br>- Why and how those farming practices improve animal welfare? Why are those practices not applied in your country? Are laws and regulations antagonistic to those practices? Or is it due to voluntary standards applied in your country?<br>- Why and how farming practices reduce animal welfare? Why are those practices applied in your country? Are laws and regulations allowing or even encouraging those practices? Or is it due to voluntary standards applied in your country?<br>- If the person talks only about a particular point, try to cover all different aspects (please refer to the ATOL and EOL ontology for the description of those different aspect). For example: What

		about feeding, what kind of food have you noticed have impact on animal's behaviour?<br>- What are the levers that could promote the adoption of practices which improve animal welfare? What are the levers that could promote the elimination of practices which impact negatively animal welfare? (These can be technical, ethical, social economic, policy or other levers)<br>- What factors prevent the actors from improving animal welfare? Labour needs? Financial constraints? Technical issues? Regulations? Other factors?
**Topic 2** : Assessment of animal welfare (10 minutes)	- How do you assess animal welfare in your farm (*or in farms you work with*)?<br>- What kind of useful and non-useful information does animal welfare assessment produce?<br>- What kind of information are you missing to assess animal welfare?	- Which tools and parameters do you use (*or witness to be used by farmers*) to assess animal welfare and why? If there are tools or parameters that you have decided not to use, what are their disadvantages compared to another tool?<br>- What information do you need to assess animal welfare on your herd?<br>- What kind of information do these tools provide? How do you interpret this information?<br>- What information are you missing to better assess your animal welfare? (*.. are missing to get a better assessment for the welfare of your customer's animal?*)
**Topic 3** : Impact of other actors (10 minutes)	- Which actors (in addition to farmers) have a major impact on animal welfare in outdoor or low-input organic poultry and pig production systems?<br>- Why are they important actors in this respect?<br>- Are you aware of any public or private sector initiatives (national or European) or emerging discussion which aim to improve animal welfare in outdoor or low-input organic poultry and pig production systems or criticise animal welfare in these systems? How would these initiatives influence farming and farmers' welfare (One welfare approach)?	- Which emerging arguments do you see in discussion concerning animal welfare in your country or in Europe?<br>- What are the customers/citizens points of view concerning animal welfare?<br>- What actions the government or the European Union is taking? What else could they do?<br>- What do you know about other actors' point of view? Which arguments are they using? What do you think about these arguments?<br>- How could the farming respond to these initiatives in the short-term and beyond?<br>- Do these initiatives and practices affect environmental impacts of outdoor or low-input organic poultry and pig production systems?<br>- How about your welfare? Will these initiatives affect your workload? Your lifestyle? Your feelings toward the animals?
**Closing question(s)** (5 minutes)	Can you please summarise up to ten most crucial points that you think impacts on (species the farmers is dealing with)'s animal welfare in low-input outdoor or organic production systems?	- What are the main benefits and disadvantages of organic and low-input outdoor pig and poultry farms (layers, broilers or pigs) in your country when animal welfare is concerned?

		- Can you suggest any key documents or studies that are addressing these concerns, barriers and levers in your country?

B- Results: Main issues in laying hens in organic and free range production according to key informants

The main issues mentioned in laying hen production were: issues related to biosecurity, lack of range use, range management and feeding (in 4 countries); issues related to feather pecking, coping with weather, regulation, flock size or density and predation (in 3 countries); bone fractures and lack of robustness (in 2 countries); parasitism, nervousness, water quality and time spent by farmers (in 1 country).

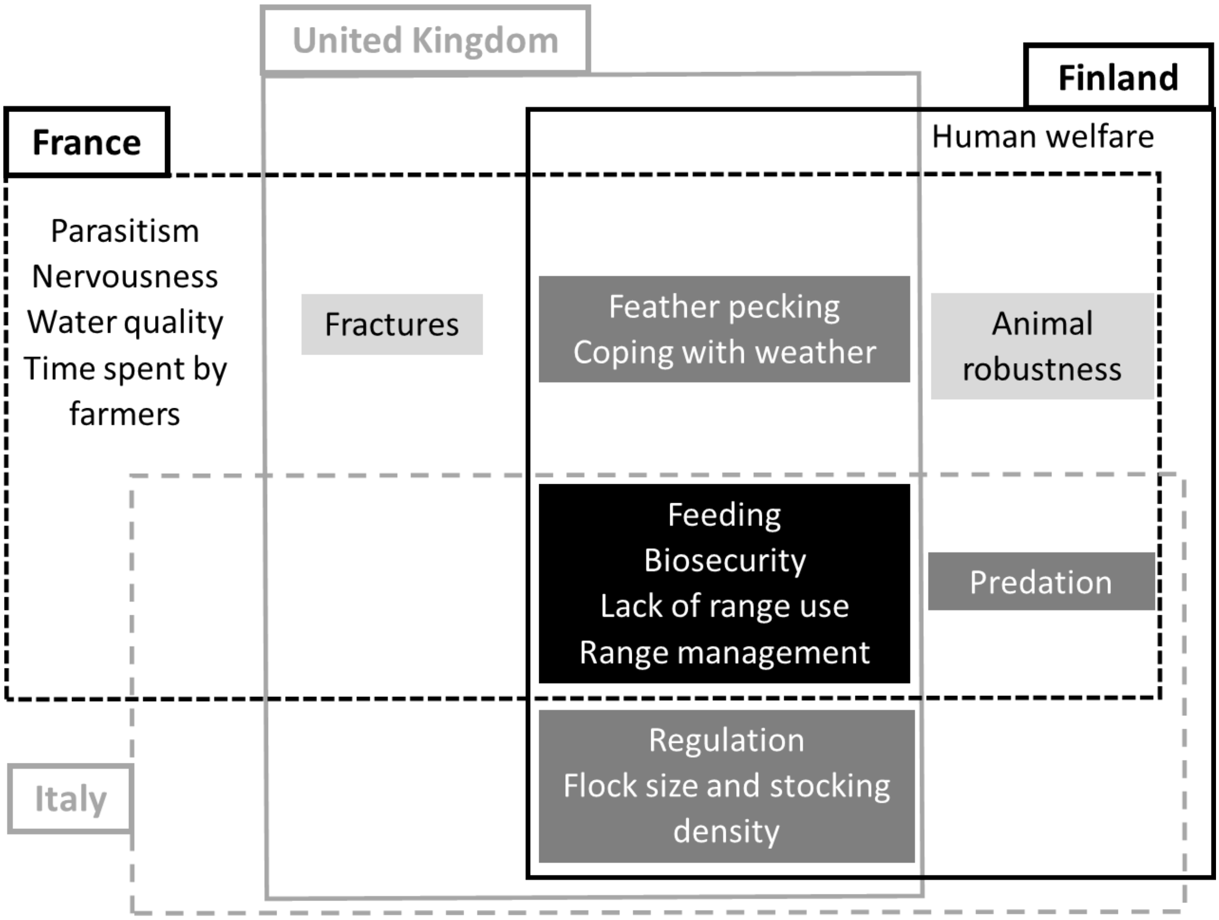

Supplementary data S3

Description of the health and behavioural activities looked into according to ATOL database (Animal Trait Ontology for Livestock; http://www.atol-ontology.com)

Traits	ATOL ref	Description
susceptibility to bacterial infection	ATOL_0001570	any detectable or measurable disorder, or clinical effect reflecting the degree of pathogenicity or lethality induced in animals or animal populations by bacterial invasion, bacterial components or bacterial productions
susceptibility to viral infection	ATOL_0000408	any detectable or measurable disorder, or clinical effect reflecting the degree of pathogenicity or lethality induced in animals or animal populations by viral invasion and multiplication
parasitic infestation response trait	ATOL_0001571	any measurable or observable characteristic related to the ability of an animal to react to parasitic infestation
helminthic infestation response	ATOL_0001573	any measurable or observable characteristic related to the ability of an animal to react to infestation of parasitic worms
investigative behaviour trait	ATOL_0000844	any measurable or observable characteristic related to the behaviour devoted to investigate the environment (physical or social), expressed by motor activities such as sniffing, pecking, scratching, licking, biting, looking at
feather pecking	ATOL_0000098	any measurable or observable characteristic related to the pecking of the other birds in an enclosure, conspecific inducing deep lesions on the body and potentially inducing the death of the receiver
